# Supplementary material for: Characterization and Potential Action Mode Divergences of Homologous ACO1 Genes during the Organ Development and Ripening Process between Non-Climacteric Grape and Climacteric Peach
Source: Int J Mol Sci. 2024 Jan 8;25(2):789. doi: 10.3390/ijms25020789 (PMC10815418; doi:10.3390/ijms25020789)
Supplement: Supplementary file 1 [file ijms-25-00789-s001.zip › Figure S2.pdf]

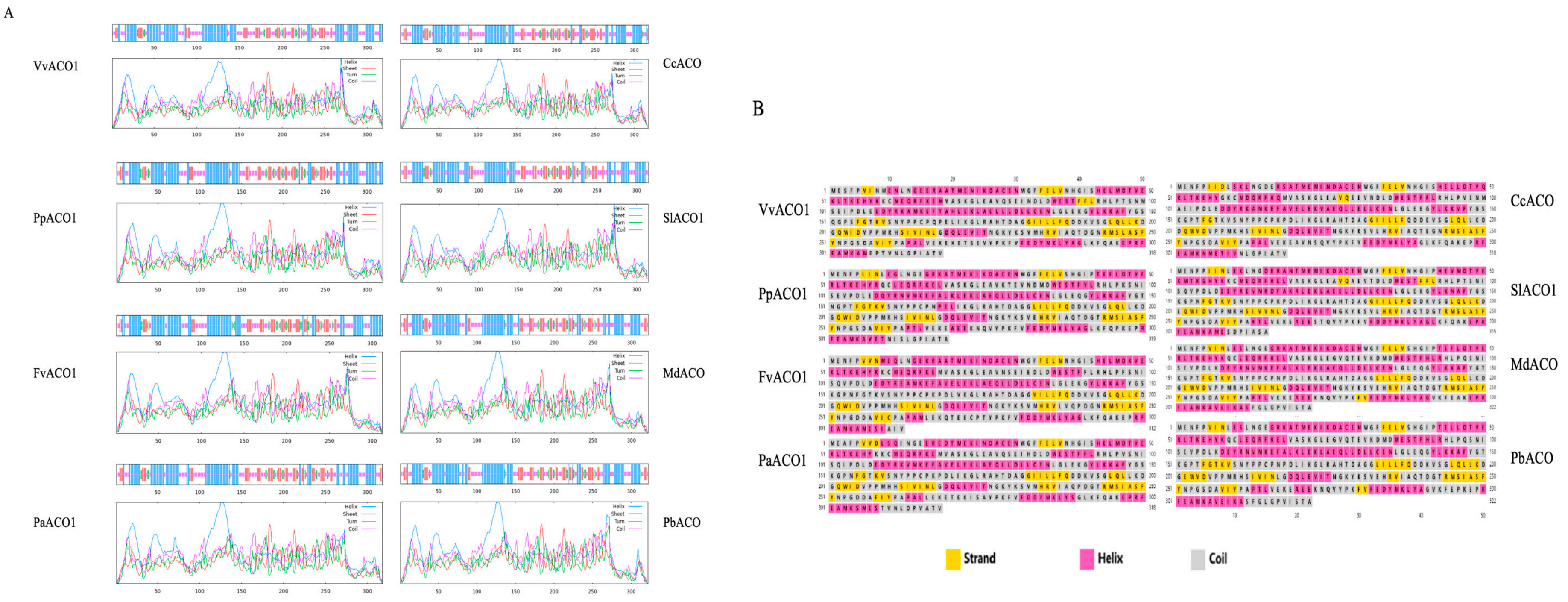

Figure S2. (**A**) The secondary structures of homologours ACO1 across 8 plant species using the PRABI（https://npsa-pbil.ibcp.fr/cgi-bin/secpred_sopma.pl）program; (**B**) The secondary structures of homologours ACO1 across 8 plant species using the PSIPRED (http://bioinf.cs.ucl.ac.uk/psipred/) program
